# Quality of death and end-of-life care among stroke patients: A comparative study of Mexican American and non-hispanic white surrogate decision makers

**DOI:** 10.1016/j.neuros.2025.100015

**Published:** 2025-11-02

**Authors:** Imadeddin Hijazi, Lewis B Morgenstern, Robert Michael Miller, Erin Case, Madeline Kwicklis, Darin B Zahuranec

**Affiliations:** a Stroke Program, Department of Neurology, University of Michigan Medical School, United States; b Center for Social Epidemiology and Population Health, Department of Epidemiology, University of Michigan School of Public Health, United States

**Keywords:** Stroke, End-of-life care, Mexican American, Hispanic/Latinx

## Abstract

**Background:**

Racial and ethnic differences in patterns of end-of-life care have been previously reported, though there has been little work on the quality of end-of-life care in Mexican American (MA) stroke patients.

**Purpose:**

To compare surrogate decision maker reports of quality of death and end-of-life care among deceased MA and non-Hispanic White (NHW) stroke patients.

**Methods:**

Stroke patients and their surrogate decision makers were identified from an ongoing population-based cohort study in Nueces County, Texas, USA. Surrogates completed the Quality of End-of-Life Care (QEOLC, primary outcome) and the Quality of Death and Dying (QODD-1, secondary outcome) measures, both ranging 0–10 (10=better). Linear regression models with generalized estimating equations were used to assess ethnic differences in outcomes, adjusted for pre-specified covariates.

**Results:**

A total of 138 surrogates were enrolled for 113 deceased stroke patients (MA = 67, NHW = 46; median patient age 78 years; female: 53%; died in the hospital: 51%). Surrogates’ median age was 60 years, and 75% were women. The overall QEOLC (Median=8.5; IQR 8,10) and QODD-1 scores (Median=10, IQR 8,10) were high. There was no ethnic difference on the QEOLC (0.03 ± 0.46, p = 0.95) or QODD-1 (−0.60 ± 0.54, p = 0.27) after adjustment.

**Conclusions:**

Surrogates in this community reported high quality of end-of-life care and death after stroke with no differences by ethnicity. The lack of disparity is a welcome finding, though more work in communities with lower acculturation and considering other aspects of end-of-life care is needed.

## Introduction

End-of-life care is a critical aspect of patient care that ideally focuses on the comfort, dignity, and respect afforded to patients during their final days. However, prior studies have shown racial and ethnic disparities in end-of-life care, with historically underrepresented groups generally reported to have less use of do-not-resuscitate (DNR) orders and greater intensity of treatment when compared with non-Hispanic white (NHW) patients [1–3]. Social determinants of health, including structural and systemic racism, likely explain these differences in outcomes, though precise mechanisms in the context of inequities in end-of-life care remain to be fully understood [4].

For stroke patients, the end-of-life period can be especially challenging. The sudden onset of significant disability and cognitive decline following stroke can complicate family surrogate decision making at the end-of-life [5–6]. In the context of these difficulties, understanding how different racial and ethnic groups perceive the quality of end-of-life care is essential. Quality of death and quality of end-of-life care are emerging measures of clinician and health system performance that assess the care provided to dying patients as reported by family members and surrogate decision makers. To date, few studies have specifically focused on the Mexican American (MA) patient population at the end of life, who make up about 60 % of the overall Hispanic/Latin(a,e,o,x) (H/L) population in the United States, with current projections indicating that H/L population will continue to rapidly increase through 2060 [7].

The primary objective of this study is to investigate the quality of death and end-of-life care as reported by MA surrogates and NHW surrogates of stroke patients. We hypothesized that surrogates of deceased MA stroke patients would report lower quality of death and end-of-life care compared to those of NHW stroke patients. This hypothesis is grounded in existing research suggesting that historically underrepresented racial and ethnic minority groups overall experience worse outcomes, which may stem from poorer healthcare access [8–9] and in-equitable administration of treatments [10].

## Methods

### Study design and participant eligibility

This prospective cohort study recruited participants from two studies: the Brain Attack Surveillance in Corpus Christi (BASIC) and Outcomes Among Surrogate Decision Makers In Stroke (OASIS) studies, both of which focus on stroke outcomes in Nueces County, Texas. Their methods have been previously described [11–12]. Nueces County is located on the coast of the Gulf of Mexico, with about 63 % of the population identifying as MA and about 30 % identifying as NHW [13]. This analysis was a primary pre-specified hypothesis of the OASIS study (NIH grant R01NS091112). Individuals (aged ≥45 years) with ischemic stroke or intracerebral hemorrhage were identified via active and passive surveillance [12]. Patients or a proxy were invited to participate in an interviewer-administered quantitative survey for the parent BASIC study, with initial contact delayed for at least 4 weeks in case of patient death. After the BASIC survey, participants were screened for the presence of a surrogate decision maker. Surrogate decision makers were eligible for OASIS if they were ≥18 years old, able to communicate in English or Spanish, served as a surrogate decision maker for a BAISC-enrolled patient, and self-identified as being significantly involved in discussions with the health care team about life-sustaining treatments (do-not-resuscitate orders, feeding tube, mechanical ventilation, or transition to comfort care/palliative care/hospice). Dyads were eligible for the current manuscript if the patient died before the time of the OA-SIS post-discharge survey and the patient was of MA or NHW race and ethnicity (other races and ethnicities excluded due to low numbers). Interviews were conducted in English or Spanish based on respondent preference. All participants (or an authorized representative) gave written or verbal informed consent.

### Outcome measures and covariates

The primary outcome was the validated family 10-item Quality of End-of-Life Care scale (QEOLC) [14–15]. which measures the quality of care received at the end of life, focusing on aspects such as communication, symptom management, and emotional support. A total QEOLC score was calculated for each surrogate based on the average of the completed items. Response options included “NA” or “don’t know,” which were not included in the calculation of the total scores. The secondary outcome was the single-item Quality of Death and Dying (QODD-1), [16] which assesses the overall quality of the patient’s death (“How would you rate [patient’s] overall experience of dying and death in the last 7 days of life?”). Note that due to other survey skip patterns, the QODD-1 was only asked of individuals who died in a hospital, inpatient hospice, or other nursing or rehabilitation facility. Both instruments are scored on a 0–10 scale where higher scores indicate better perceived quality of care or death.

Covariates collected via self-report included surrogate age, sex, patient relationship to the surrogate (categorized as parent, spouse, or other), education level, and country of birth. Race and ethnicity were collected via self-report, though patient race and ethnicity were supplemented with the medical record in the event of missing data. The primary exposure was patient ethnicity; thus, terms such as “MA surrogates” will be used to describe surrogates of MA patients. Surrogates also self-reported health literacy using a validated single-item measure (“How comfortable are you filling out medical forms by yourself?” with responses ranging from “Not at all” to “Extremely”) [17]. Acculturation was assessed with the 4-item brief acculturation scale for Hispanics [18]. These items asses language usage in a variety of settings with five response options ranging from 1= “Only Spanish” to 5= “Only English”, with scores averaged across all items and a mean score >2.99 indicating high acculturation. Other covariates were abstracted from the medical record, including stroke type (ischemic vs. hemorrhagic), initial National Institutes of Health Stroke Scale (NIHSS), [19] intensive care unit (ICU) admission status (yes or no), and health insurance (yes or no).

### Statistical analysis

Descriptive characteristics of patients and surrogates were summarized overall and compared by patient ethnicity. P-values were calculated using a chi-square test or a Fisher’s exact test for categorical variables, and a *t*-test assuming equal variance for the continuous variables. To account for missing covariate data, multivariate imputations by chained equations were used to impute the data and account for statistical uncertainty in the imputations [20]. The results were then pooled. Imputed data sets were used for the primary analysis, though a complete case analysis was also done as a sensitivity analysis. The primary and secondary outcomes were analyzed using linear regression models and generalized estimating equations to account for the potential clustering of responses within families. Model covariates were pre-specified on theoretical or known association with ethnicity and/or outcomes [21–24]. Sequential linear regression models were applied to examine the impact of patient ethnicity (primary exposure) on the primary and secondary outcomes. Model 0 included patient ethnicity (MA vs. NHW); Model 1 added patient age, patient sex, and surrogate relationship; Model 2 added clinical factors: NIHSS, ICU admission, and stroke type (intracerebral hemorrhage vs. ischemic); Model 3 added patient insurance (yes/no) and patient/surrogate education (more than high school or not); Model 4 added time from stroke to death and time from death to survey.

## Results

Between April 2016 and September 2020, a total 143 surrogates completed a post-discharge survey for 116 patients. One surrogate had completely missing QEOLC data so was excluded, and one surrogate corresponded to two patients and was also excluded. After excluding 3 more surrogates for identifying patients’ race or ethnicity as other than MA or NHW, a total of 138 surrogates for 113 MA or NHW stroke patients were included in the analytic sample. We have previously reported that ~45 % of OASIS patient participants were deceased at the time of the discharge survey, with no difference among eligible patients in demographics, stroke type, severity, or in-hospital mortality between those enrolling and not enrolling in OASIS [23].

Patient characteristics are shown in Table 1. Surrogate characteristics are shown in Table 2. Notably, most surrogate decision makers were female and children of the patient. Surrogates of MA patients were on average younger (58 years old versus 61 years old) and were less likely to have completed education beyond high school (19.8 % versus 40.4 %). MA patients were less likely than NHW patients to have completed education beyond high school (27.3 % versus 58.7 %) and were more likely to be insured (95.5 % of MA patients were insured, whereas only 89.1 % of NHW patients were insured). Most interviews (>95 %) were conducted in English in each ethnic group. Most surrogates of MA patients were born in the United States (89 %), and over 90 % reported high scores on the brief acculturation scale. Both groups reported high health literacy, but surrogates of MA patients were more likely to report high health literacy than those of NHW patients (90.12 % MA versus 73.68 % NHW).

Responses to individual items on the QEOLC and QODD-1, including the proportion of valid numeric responses used in the total score calculation, are summarized in Table 3. The overall QEOLC scores were high (Median=8.5; IQR 8,10), as were the QODD-1 scores (Median=10, IQR 8,10), in both MA and NHW surrogates. Supplemental Figure 1 shows that 74 % of surrogates provided a valid numeric response to at least 7 of 10 QEOLC items. QEOLC item 1 (“Talks to [patient] in an honest and straightforward way”) had the lowest proportion of numeric responses, followed by item 10 (“Makes [patient] feel confident that he/she will not be abandoned prior to death”), and then item 3 “Responsive to [patient’s] emotional needs”.

In the sequential modeling for QEOLC, there was no difference between MA and NHW surrogates when unadjusted (Beta=−0.13, SE=0.46, p = 0.78), in any of the sequential regression models (Supplemental Table S1 and S2), nor in the fully adjusted model (Beta=−0.60, SE=0.54, *p* = 0.27, see Table 4). None of the other included covariates were associated with QEOLC in the final fully adjusted model. The complete case analysis showed similar results to the imputed analysis (Supplemental Table S2).

For the QODD-1, unadjusted analyses revealed that surrogates of MAs reported lower scores by about 1 point when compared to NHWs (Beta=−0.99, SE=0.48, p = 0.04, see Supplemental Tables 3 and 4]). However, this difference was attenuated and no longer statistically significant when adjusted for patient age, sex, and surrogate relationship (Beta=−0.692; SE=0.478; p = 0.147 [Model 1 in Supplemental Table S3]), or in the final fully adjusted model (Beta=0.03, SE=0.46, p = 0.95).

## Discussion

Contrary to our original hypothesis, we did not identify an ethnic difference in surrogate reports of the quality of death and end-of-life care after stroke between MA patients and NHW patients after adjusting for demographic and clinical factors. This result is encouraging, as it implies that in this community, ethnic disparities in end-of-life care may not be as pronounced as reported in other populations [25–27]. Furthermore, the high scores on both surveys indicate overall satisfaction with care regardless of ethnicity.

One explanation for our findings’ contrast to previous research is that prior papers mostly focused on palliative care utilization and rates of rehospitalization near the time of death as indicators of quality of end-of-life care, rather than surrogates’ self-report of quality of end-of-life care. It is possible that these objective disparities do not affect surrogates’ subjective experience with the quality of death. One of the few studies that reports a modest negative difference between the perception of care of surrogates of H/L and NHW patients surveyed the family members of Veterans Affairs patients in a single hospital [28]. Our present study, on the other hand, specifically examined death after stroke in community hospitals.

Moreover, one crucial difference in this community of Corpus Christi is that the majority of the population identifies as having MA origin. Other studies have not specifically examined MA-majority communities. The surrogates of MA stroke patients in our present study also report relatively high acculturation, high health literacy, and preferred English as the primary language for the interview. These characteristics suggest that the surrogates of MA patients in this community are highly culturally integrated, which may further explain our finding of no difference between the groups. Thus, our results may not apply to other communities where the MA community has a higher proportion of primarily Spanish-speaking or foreign-born individuals or lower acculturation.

The QEOLC had several items with a high proportion of surrogates unable to provide a numeric response. While we did not conduct formal analysis, the most commonly occurring items that surrogates responded “Don’t know/NA” tended to involve constructs assessing how the physician communicated with the patient directly. The inability of some surrogates to answer questions regarding communication between physicians and patients may reflect the patients’ mental status or inability to communicate. However, those who did answer these questions largely viewed their care favorably. The highest scores were for items regarding physician-surrogate communication and acknowledgement of patients’ physical needs at the end of life. In general, it appears that this surrogate population felt that the physician teams were communicating with them effectively and adequately, and that their loved ones’ pain and other needs were being adequately met.

Based on our findings, several additional steps could be considered in the investigation of end-of-life care in diverse populations. First, more work should be done with larger sample sizes, with an emphasis on the inclusion of individuals with lower levels of acculturation, English proficiency, or health literacy. Larger studies could not only help to identify whether any subgroups remain at risk for poor outcomes but also could help to determine factors (e.g., religion, faith, culture, social support, education, health literacy) that are protective among at-risk individuals. Protective factors could then be explored as potential intervention mechanistic targets to support optimal outcomes in end-of-life care. Future studies should also consider other outcome measures, such as patient symptom burden or bereaved family member psychological outcomes (e.g., prolonged grief), to determine if the encouraging findings seen in our current work extend to other aspects of end-of-life care.

## Limitations

Based on our modest sample size, low power (type 2 error) could also explain our findings. However, the beta coefficient for ethnicity on the QEOLC was quite small at 0.03 (scale range 0–10), suggesting that it is unlikely that a difference would have been seen even with larger samples. Other limitations of this study include the potential for response bias, as participation was voluntary. To mitigate this, we attempted to recruit all surrogates and incentivized participation. Additionally, the reliance on surrogate reports may not fully capture the complexities of patient experiences at the end of life. Based on our sample size, we could not draw meaningful conclusions about the small number of individuals in our study who were uninsured, preferred Spanish for the interviews, reported low acculturation, or were born in Mexico. This was a single-county investigation with a Hispanic population with high acculturation, and our results may not generalize to other communities with lower acculturation. We did not have data on interpreter use or language concordance from the hospitalization, though most surrogates were comfortable communicating in English.

## Conclusion

In conclusion, our study did not identify differences in the quality of death or end-of-life care between MA and NHW surrogates in this highly acculturated Hispanic-majority community. These results are encouraging, though further study is needed in larger samples and in other settings to determine if these findings are generalizable to other communities with a different cultural population mix.

## Supplementary Material

1

2

Supplementary materials

Supplementary material associated with this article can be found, in the online version, at https://doi.org/10.1016/j.neuros.2025.100015.

## Figures and Tables

**Table 1:** Patient characteristics.

Characteristic	All N ( %) or median (IQR)	Mexican American N ( %) or median (IQR)	Non-Hispanic White N ( %) or median (IQR)	p-value*

N ( %)	N = 113 (100.00 %)	N = 67 (59.3 %)	N = 46 (40.7 %)	
Age	78.00 (66, 89)	74.00 (65.50, 87.00)	86.50 (67.25, 91)	0.212
Female sex	60 (53.10 %)	36 (53.7 %)	24 (52.2 %)	1
Stroke Type				
Ischemic	82 (72.57 %)	47 (70.2 %)	35 (76.1 %)	0.631
Intracerebral hemorrhage	31 (27.43 %)	20 (29.9 %)	11 (23.9 %)	0.631
National Institutes of Health Stroke Scale Score	18.00 (10.00, 27.00)	19.00 (10.5, 29.5)	18.00 (10.00, 25.75)	0.334
ICU admission (yes)	65 (60.75 %)	37 (59.0 %)	29 (63.0 %)	0.649
Days survived	7 (3, 13)	7 (3, 13)	6 (3, 12)	0.882
Health insurance (yes)	105 (92.92 %)	64 (95.5 %)	41 (89.1 %)	0.006
Education				
High school or less	67 (59.82 %)	48 (72.7 %)	19 (41.3 %)	0.002
Beyond high school	45 (40.18 %)	18 (27.3 %)	27 (58.7 %)	0.002

* P-values compare Mexican American and non-Hispanic White individuals.

**Table 2:** Surrogate characteristics.

Characteristic	All N ( %) or median (IQR)	Mexican American patients N ( %) or median (IQR)	Non-Hispanic White patients N ( %) or median (IQR)	p-value*

N ( %)	N = 138 (100.00 %)	N = 81 (58.70 %)	N = 57 (41.30 %)	
Age	60.00 (52.00, 68.00)	58.00 (48.00, 66.00)	61.00 (56.00, 70.00)	0.008
Female sex	103 (74.64 %)	63 (77.78 %)	40 (70.18 %)	0.417
Relationship (surrogate perspective)				
Patient as Parent	90 (65.22 %)	56 (69.14 %)	34 (59.65 %)	0.019
Patient as Spouse	30 (21.74 %)	12 (14.82 %o)	18 (31.58 %)	0.019
Other	18 (13.04 %)	13 (16.04 %)	9 (8.77 %)	0.019
Surrogate Education				
High school or less	99 (71.74 %)	65 (80.24 %)	34 (59.65 %)	0.029
Beyond high school	39 (28.26 %)	16 (19.75 %)	23 (40.35 %)	0.029
Interview language^†^ (English, vs. Spanish)	136 (98.55 %)	79 (97.53 %)	57 (100 %)	0.512
Country of birth^†^ (United States)	126 (91.3 %)	72 (88.89 %)	54 (94.74 %)	0.359
Brief acculturation scale				
Mean (SD)	4.37 (0.81)	3.95 (0.84)	4.96 (0.10)	<0.001
High Acculturation^†^, N ( %)	131 (94.93 %)	74 (91.36 %)	57 (100 %)	0.041
High Health literacy* (extremely or quite a bit confident in filling out medical forms)	115 (83.33 %)	73 (90.12 %)	42 (73.68 %)	0.019

Abbreviations: IQR=interquartile range.

* P-values compare Mexican Americans and non-Hispanic White individuals.

† P-values were calculated using Fisher’s exact test due to the low expected cell counts.

**Table 3:** Individual item responses for QEOLC (N = 138) and QODD-1 (N = 120*).

Item question	Median	IQR	% Valid Responses

QEOLC 1 - Talks to [patient] in an honest and straightforward way	7	5,10	46.4
QEOLC 2 - Responsive to [patient]’s emotional needs	8	5,10	60.1
QEOLC 3 - Helps you and [patient] get consistent information from the entire healthcare team	8	5,10	94.2
QEOLC 4 - Takes into account [patient’s] wishes when treating pain and symptoms	9	7,10	82.6
QEOLC 5 - Admits when he or she (the clinician) doesn’t know something	8	5,10	63.0
QEOLC 6 - Treats the whole person, not just the disease	9	5,10	94.2
QEOLC 7 - Knowledgeable about the care [patient] needs during the dying process	9	7,10	96.4
QEOLC 8 - Openly and willingly communicates with you	9	7,10	97.8
QEOLC 9 - Acknowledges and respects [patient’s] personal beliefs	10	6,10	81.9
QEOLC 10 - Makes [patient] feel confident that he/she will not be abandoned prior to death	9	5,10	59.4
QODD-1 - How would you rate X’s overall experience of dying and death in the last 7 days of life?*	10	8,10	95.0

Abbreviations: IQR=interquartile range; QEOLC= Quality of End-of-Life Care; QODD= Quality of Death and Dying.

* The QODD-1 was only asked when patients died in a healthcare facility. Thus, the proportion of valid numeric responses for the QODD is 114/120.

**Table 4:** Fully adjusted linear regression model results for QEOLC and QODD-1.

	Quality of End-of-Life Care (QEOLC)			Quality of Death and Dying (QODD-1*)		
	*N* = 138 Surrogates			*N* = 114 Surrogates		
Covariate	Beta	Standard Error	P	Beta	Standard Error	P

Patient race and ethnicity (MA vs. NHW)^†^	0.03	0.46	0.95	−0.60	0.54	0.27
Patient age	0.03	0.02	0.24	0.03	0.03	0.44
Patient female (vs. male)	−0.02	0.43	0.97	0.31	0.54	0.57
Relationship to surrogate						
Parent	−0.58	0.50	0.25	−0.86	0.58	0.14
Other	−0.48	0.62	0.43	−1.39	1.12	0.22
Spouse (Reference)	–	–	–	–	–	–
NIHSS, per point	−0.02	0.02	0.45	0.002	0.03	0.94
ICU admission (yes vs. no)	−0.06	0.52	0.91	0.41	0.65	0.53
Intracerebral hemorrhage (vs. ischemic stroke)	0.15	0.53	0.78	0.49	0.62	0.43
Patient education beyond high school (yes vs. no)	0.49	0.50	0.33	−0.03	0.51	0.96
Patient Insurance (yes vs. no)	0.18	0.66	0.79	−0.97	0.84	0.25
Surrogate education beyond high school	−0.59	0.48	0.22	0.66	0.65	0.31
Days from stroke to death	−0.003	0.008	0.74	−0.005	0.01	0.67
Days from death to survey	−0.007	0.006	0.22	−0.01	0.007	0.07

Abbreviations: MA=Mexican American; NHW=non-Hispanic White; NIHSS=National Institutes of Health Stroke Scale; ICU= Intensive Care unit; QEOLC= Quality of End-Of-Life Care; QODD= Quality of Death and Dying.

* QODD-1 sample is 114 surrogates, as this was only asked when patients died in a healthcare facility.

† Patient race and ethnicity were based on self-report of patient or proxy, supplemented by medical record if self-report was not available. Other race and ethnicity were excluded due to low numbers.

## Data Availability

The data that support the findings of this study are available from the corresponding author, DZ, upon reasonable request.
