# Supplementary material for: Quality of death and end-of-life care among stroke patients: A comparative study of Mexican American and non-hispanic white surrogate decision makers
Source: Equity Neurosci. Author manuscript; Available in PMC 2025 Dec 13. (PMC12700226; doi:10.1016/j.neuros.2025.100015)

**Supplemental Table S1: QEOLC-10 Complete Case**

|  | Model 0: unadjusted | | Model 1: adjusted for prespecified demographics | | Model 2: adjusted for prespecified demographics and clinical variables | | Model 3:  adjusted for prespecified demographics and clinical variables | | Model 4: fully adjusted | |
| --- | --- | --- | --- | --- | --- | --- | --- | --- | --- | --- |
| Characteristic | Beta  (SE) | P | Beta  (SE) | P | Beta  (SE) | P | Beta  (SE) | P | Beta  (SE) | P |
| Patient Ethnicity  (MA) | -0.13  (0.457) | 0.775 | 0.075  (0.449) | 0.87 | -0.12  (0.468) | 0.8 | -0.04  (0.492) | 0.935 | -0.161  (0.494) | 0.74 |
| Patient Age (Years) |  |  | 0.02  (0.019) | 0.28 | 0.025  (0.02) | 0.21 | -0.03  (0.02) | 0.16 | 0.029  (0.022) | 0.19 |
| Patient sex  (Female) |  |  | -0.064  (0.437) | 0.89 | -0.279  (0.436) | 0.52 | -0.301  (0.428) | 0.48 | -0.212  (0.428) | 0.62 |
| Parent v Spouse |  |  | -0.852  (0.512) | 0.1 | -0.655  (0.62) | 0.29 | -0.591  (0.599) | 0.32 | -0.467  (0.628) | 0.46 |
| Spouse v Other |  |  | -0.756  (0.573) | 0.19 | -0.716  (0.644) | 0.27 | -0.642  (0.668) | 0.34 | -0.495  (0.718) | 0.49 |
| NIHSS |  |  |  |  | -0.028  (0.024) | 0.24 | -0.025  (0.024) | 0.31 | -0.023  (0.024) | 0.34 |
| ICU admit (yes) |  |  |  |  | 0.009  (0.527) | 0.99 | -0.109  (0.524) | 0.84 | -0.051  (0.534) | 0.93 |
| Stroke type (intracerebral hemorrhage) |  |  |  |  | 0.348  (0.551) | 0.53 | 0.364  (0.543) | 0.50 | 0.304  (0.548) | 0.58 |
| Patient Education (> High school) |  |  |  |  |  |  | 0.587  (0.541) | 0.28 | 0.602  (0.527) | 0.25 |
| Patient insured (Yes) |  |  |  |  |  |  | -0.091  (0.660) | 0.89 | 0.009  (0.667) | 0.99 |
| Surrogate education (> High school) |  |  |  |  |  |  | -0.578  (0.503) | 0.25 | -0.511  (0.517) | 0.32 |
| Days survived |  |  |  |  |  |  |  |  | -0.001  (0.009) | 0.88 |
| Time death to survey |  |  |  |  |  |  |  |  | -0.007  (0.006) | 0.27 |

**Supplemental Table S2: QEOLC-10 Imputed Analysis**

|  | Model 0:  unadjusted | | Model 1:  adjusted for prespecified demographics | | Model 2:  adjusted for prespecified demographics and clinical variables | | Model 3:  adjusted for prespecified demographics and clinical variables | | Model 4:  fully adjusted | |
| --- | --- | --- | --- | --- | --- | --- | --- | --- | --- | --- |
| Characteristic | Beta  (SE) | P | Beta  (SE) | P | Beta  (SE) | P | Beta  (SE) | P | Beta  (SE) | P |
| Patient Ethnicity  (MA) | -0.130  (0.457) | 0.78 | 0.075  (0.449) | 0.87 | 0.094  (0.446) | 0.83 | 0.112  (0.465) | 0.846 | 0.030  (0.463) | 0.95 |
| Patient Age (Years) |  |  | 0.02  (0.019) | 0.28 | 0.022  (0.019) | 0.26 | 0.025  (0.022) | 0.225 | 0.025  (0.021) | 0.24 |
| Patient sex  (Female) |  |  | -0.064  (0.437) | 0.89 | -0.123  (0.429) | 0.78 | -0.087  (0.426) | 0.57 | -0.016  (0.427) | 0.97 |
| Parent v Spouse |  |  | -0.852  (0.512) | 0.1 | -0.717  (0.523) | 0.17 | -0.652  (0.497) | 0.174 | -0.580  (0.501) | 0.25 |
| Spouse v Other |  |  | -0.756  (0.573) | 0.19 | -0.689  (0.583) | 0.24 | -0.601  (0.598) | 0.314 | -0.477  (0.619) | 0.44 |
| NIHSS |  |  |  |  | -0.025  (0.024) | 0.29 | -0.023  (0.024) | 0.336 | -0.019  (0.024) | 0.44 |
| ICU admit (yes) |  |  |  |  | 0.004  (0.510) | 0.99 | -0.109  (0.512) | 0.982 | -0.057  (0.520) | 0.91 |
| Stroke type (intracerebral hemorrhage) |  |  |  |  | 0.231  (0.529) | 0.66 | 0.242  (0.522) | 0.645 | 0.145  (0.529) | 0.78 |
| Patient Education (> High school) |  |  |  |  |  |  | 0.443  (0.513) | 0.871 | 0.490  (0.504) | 0.33 |
| Patient insured (Yes) |  |  |  |  |  |  | 0.111  (0.654) | 0.339 | 0.179  (0.663) | 0.79 |
| Surrogate education (> High school) |  |  |  |  |  |  | -0.629  (0.470) | 0.18 | -0.588  (0.945) | 0.22 |
| Days survived |  |  |  |  |  |  |  |  | -0.003  (0.008) | 0.74 |
| Time death to survey |  |  |  |  |  |  |  |  | -0.007  (0.006) | 0.22 |

Abbreviations: MA= Mexican American; NIHSS=National Institutes of Health Stroke Scale; SE=Robustness/Standard error

**Supplemental Table S3: QODD-1 Complete Case**

|  | Model 0:  unadjusted | | Model 1:  adjusted for prespecified demographics | | Model 2:  adjusted for prespecified demographics and clinical variables | | Model 3:  adjusted for prespecified demographics and clinical variables | | Model 4:  fully adjusted | |
| --- | --- | --- | --- | --- | --- | --- | --- | --- | --- | --- |
| Characteristic | Beta  (SE) | P | Beta  (SE) | P | Beta  (SE) | P | Beta  (SE) | P | Beta  (SE) | P |
| Patient Ethnicity  (MA) | -0.994  (0.478) | 0.04 | -0.692  (0.478) | 0.15 | -0.869  (0.583) | 0.14 | -0.73  (0.570) | 0.20 | -0.837  (0.590) | 0.16 |
| Patient Age (Years) |  |  | 0.008  (0.025) | 0.75 | 0.02  (0.028) | 0.49 | 0.025  (0.038) | 0.50 | 0.028  (0.035) | 0.42 |
| Patient sex  (Female) |  |  | 0.238  (0.556) | 0.67 | 0.133  (0.565) | 0.81 | 0.101  (0.563) | 0.86 | 0.243  (0.564) | 0.67 |
| Parent v Spouse |  |  | -0.874  (0.599) | 0.15 | -0.875  (0.608) | 0.15 | -0.85  (0.599) | 0.16 | -0.795  (0.605) | 0.19 |
| Spouse v Other |  |  | -1.633  (1.071) | 0.13 | -1.78  (1.139) | 0.12 | -1.786  (1.118) | 0.11 | -1.397  (1.162) | 0.23 |
| NIHSS |  |  |  |  | 0.002  (0.027) | 0.95 | 0.000  (0.027) | 1.0 | 0.007  (0.027) | 0.79 |
| ICU admit (yes) |  |  |  |  | 0.189  (0.674) | 0.78 | 0.229  (0.659) | 0.73 | 0.415  (0.675) | 0.54 |
| Stroke type (intracerebral hemorrhage) |  |  |  |  | 0.808  (0.637) | 0.2 | 0.683  (0.643) | 0.29 | 0.556  (0.64) | 0.39 |
| Patient Education (> High school) |  |  |  |  |  |  | 0.05  (0.569) | 0.93 | -0.037  (0.538) | 0.95 |
| Patient insured (Yes) |  |  |  |  |  |  | -1.142  (0.894) | 0.20 | -1.054  (0.891) | 0.23 |
| Surrogate education (> High school) |  |  |  |  |  |  | 0.389  (0.736) | 0.60 | 0.503  (0.672) | 0.45 |
| Days survived |  |  |  |  |  |  |  |  | -0.007  (0.014) | 0.63 |
| Time death to survey |  |  |  |  |  |  |  |  | -0.015  (0.007) | 0.04 |

**Supplemental Table S4: QODD-1 Imputed Analysis**

|  | Model 0: unadjusted | | Model 1: adjusted for prespecified demographics | | Model 2: adjusted for prespecified demographics and clinical variables | | Model 3:  adjusted for prespecified demographics and clinical variables | | Model 4: fully adjusted | |
| --- | --- | --- | --- | --- | --- | --- | --- | --- | --- | --- |
| Characteristic | Beta  (SE) | P | Beta  (SE) | P | Beta  (SE) | P | Beta  (SE) | P | Beta  (SE) | P |
| Patient Ethnicity  (MA) | -0.994  (0.478) | 0.04 | -0.692  (0.478) | 0.15 | -0.687  (0.523) | 0.19 | -0.576  (0.509) | 0.26 | -0.594  (0.539) | 0.27 |
| Patient Age (Years) |  |  | 0.008  (0.025) | 0.75 | 0.016  (0.028) | 0.56 | 0.018  (0.034) | 0.59 | 0.026  (0.034) | 0.44 |
| Patient sex  (Female) |  |  | 0.238  (0.556) | 0.67 | 0.267  (0.552) | 0.63 | 0.241  (0.534) | 0.65 | 0.314  (0.544) | 0.57 |
| Parent v Spouse |  |  | -0.874  (0.599) | 0.15 | -0.86  (0.574) | 0.14 | -0.872  (0.572) | 0.13 | -0.86  (0.579) | 0.14 |
| Spouse v Other |  |  | -1.633  (1.071) | 0.13 | -1.661  (1.088) | 0.13 | -1.732  (1.076) | 0.11 | -1.392  (1.121) | 0.22 |
| NIHSS |  |  |  |  | -0.004  (0.027) | 0.879 | -0.007  (0.027) | 0.79 | 0.002  (0.027) | 0.93 |
| ICU admit (yes) |  |  |  |  | 0.177  (0.640) | 0.78 | 0.234  (0.624) | 0.71 | 0.422  (0.650) | 0.52 |
| Stroke type (intracerebral hemorrhage) |  |  |  |  | 0.717  (0.611) | 0.24 | 0.631  (0.613) | 0.31 | 0.479  (0.617) | 0.44 |
| Patient Education (> High school) |  |  |  |  |  |  | -0.101  (0.519) | 0.85 | -0.03  (0.511) | 0.95 |
| Patient insured (Yes) |  |  |  |  |  |  | -0.986  (0.825) | 0.24 | -0.968  (0.838) | 0.25 |
| Surrogate education (> High school) |  |  |  |  |  |  | 0.558  (0.695) | 0.42 | 0.66  (0.646) | 0.31 |
| Days survived |  |  |  |  |  |  |  |  | -0.005  (0.012) | 0.67 |
| Time death to survey |  |  |  |  |  |  |  |  | -0.013  (0.007) | 0.07 |

Abbreviations: MA= Mexican American; NIHSS=National Institutes of Health Stroke Scale; SE=Robustness/Standard error

**Supplemental Figures**

Supplemental Figure 1: Distribution of Quality of End-of-life Care (QEOLC) responses showing the number of surrogates who provided a valid numeric response included in the total score.


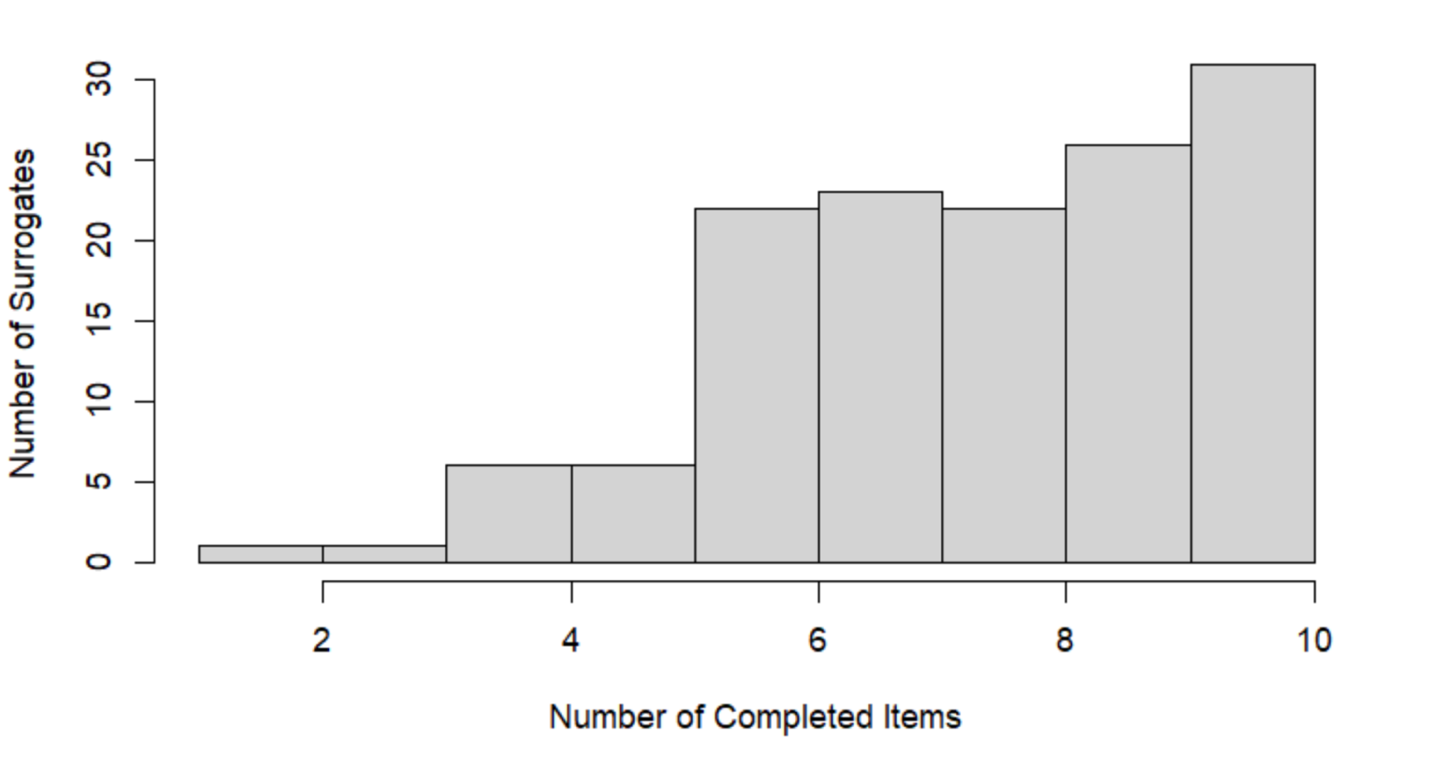

Supplement: 1 [file NIHMS2119936-supplement-1.docx]
